# Factors affecting the length of hospital stay after laparoscopic appendectomy: A single center study

**DOI:** 10.1371/journal.pone.0243575

**Published:** 2020-12-09

**Authors:** Peng Zhang, Qian Zhang, Hongwei Zhao, Yuanxin Li

**Affiliations:** Department of Gastrointestinal Surgery, Beijing Tsinghua Changgung Hospital, School of Clinical Medicine, Tsinghua University, Beijing, China; Ohio State University Wexner Medical Center Department of Surgery, UNITED STATES

## Abstract

**Aim:**

This study aimed to explore factors may affect the length of hospital stay after laparoscopic appendectomy.

**Methods:**

The data of 636 patients undergoing laparoscopic appendectomy between July 2016 and July 2019 in Beijing Tsinghua Changgung Hospital were retrospectively analyzed. The patients were divided into group A (hospital stay ≤3 days, 348 patients) and group B (hospital stay >3 days, 288 patients) according to their hospital stay.Sex, age, disease onset time(time from onset to admission), nausea, vomiting, diarrhea, peritonitis, comorbidities, and history of appendicitis; preoperative body temperature (T), white blood cell (WBC) count, percentage of neutrophilic granulocytes, and preoperative C-reactive protein (CRP) level; time from diagnosis to surgery. appendix diameter, appendicolith, and ascites in ultrasound or CT; surgical time(the surgery start time was the time of skin incision, and the end time was the time the anesthesia intubation was removed), intraoperative blood loss (the volume of blood infiltrating into a gauze was calculated by weighing the gauze infiltrated with water and calculating the volume of water), intraoperative adhesions or effusions, and stump closure methods, convert to open appendectomy, appendix pathology(perforated or gangrenous appendicitis were defined as complicated appendicitis and simple or suppurative appendicitis were defined as uncomplicated appendicitis) and antibiotic treatment schemes were analyzed.

**Results:**

Significant differences were detected between group A and group B in age (37.10 ± 13.52y vs 42.94 ± 15.57y, *P*<0.01), disease onset time (21.36 ± 16.56 h vs 32.52 ± 27.99 h, *P* <0.01), time from diagnosis to surgery (8.63 ± 7.29 h vs 10.70 ± 8.47 h, *P*<0.01); surgical time(64.09 ± 17.24 min vs 86.19 ± 39.96 min, *P* < 0.01); peritonitis(52.9% vs 74%, *P* < 0.01), comorbidities (12.4% vs 20.5%, *P* < 0.01), appendicolith (27.6% vs 41.7%, *P* < 0.01), ascites before the surgery(13.8% vs 22.9%, *P* < 0.01), intraoperative adhesions or effusions(56% vs 80.2%, *P* < 0.01); preoperative temperature (37.11 ± 0.64°C vs 37.54 ± 0.90°C, *P* < 0.01); preoperative WBC count (13.06 ± 3.39 × 109/L vs 14.21 ± 4.54 × 109/L, *P* = 0.04);preoperative CRP level(18.99 ± 31.72 mg/L vs 32.46 ± 46.68 mg/L, *P* < 0.01); appendix diameter(10.22 ± 2.59 mm vs 11.26 ± 3.23 mm, *P* < 0.01); intraoperative blood loss (9.36 ± 7.29 mL vs 13.74 ± 13.49 mL, *P* < 0.01); using Hem-o-lok for stump closure(30.7% vs 38.5%, *P* = 0.04); complicated appendicitis (9.5% vs 45.8%, *P* < 0.01); and using ertapenem for antibiotic treatment after the surgery(4.3% vs 21.5%, *P* < 0.01). Multivariate analysis demonstrated that age (OR = 1.021; 95%CI = 1.007–1.036), peritonitis (OR = 1.603; 95% CI = 1.062–2.419), preoperative WBC count (OR = 1.084; 95% CI = 1.025–1.046), preoperative CRP level (OR = 1.010; 95% CI = 1.005–1.015), time from diagnosis to surgery (OR = 1.043; 95% CI = 1.015–1.072), appendicolith (OR = 1.852; 95% CI = 1.222–2.807), complicated appendicitis (OR = 3.536; 95% CI = 2.132–5.863), surgical time (OR = 1.025; 95% CI = 1.016–1.034), use of Hem-o-lok for stump closure (OR = 1.894; 95% CI = 1.257–2.852), and use of ertapenem for antibiotic treatment (OR = 3.076; 95% CI = 1.483–6.378) were the risk factors for a prolonged hospital stay.

**Conclusions:**

The patient with appendicitis was older and had peritonitis, higher preoperative WBC count or CRP level, longer time from diagnosis to surgery, appendicolith, and complicated appendicitis, predicting a prolonged hospital stay. Shorter surgical time and the use of silk ligation for stump closure and cephalosporins + metronidazole for antibiotic treatment might be better choices to obtain a shorter hospital stay.

## Introduction

Acute appendicitis is the most common acute abdominal surgical emergency with a worldwide incidence of 6.7%-8.6% [1]. The mortality rate of acute appendicitis is low, but complications are common in patients with complex disease [2]. Despite advances in antibiotic management, surgical excision is often the first choice for treatment [3].

Laparoscopic appendectomy has become the preferred surgical method for appendicitis due to less trauma and faster recovery [4–6]. Previous studies demonstrated that the hospital stay was 1–8 days after laparoscopic appendectomy [4, 6–16]. The shorter hospital stay might lead to decreased cost and higher percentage of bed utilization. Therefore, the Chinese government established a clinical pathway and limited the hospital stay to 3 days. The purpose of this study was to explore influencing factors for a hospital stay.

## Materials and methods

The study was conducted in accordance with the principles of the Declaration of Helsinki, and the study protocol was approved by the ethics committee of Beijing Tsinghua Changgung Hospital (20277-0-01). Because of the retrospective nature of the study, patient consent for inclusion was waived.

This was a retrospective study of patients with acute appendicitis who underwent laparoscopic appendectomy between July 2016 and July 2019 at the Beijing Tsinghua Changgung Hospital. Data of 636 patients who underwent laparoscopic appendectomy between July 2016 and July 2019 were retrospectively reviewed.

The inclusion criteria were as follows: (1) patients diagnosed with acute appendicitis, (2) patients undergoing laparoscopic appendectomy, and (3) patients aged more than 15 years.

The exclusion criteria were as follows: (1) patients diagnosed with periappendicular abscess, because we followed the 2017 WESE guidelines for appendticitis treatment and choose non-operative management with antibiotics or percutaneous drainage as the first choice for it, (2) aged less than 15 years or more than 80 years, and (3) negative explorations.

The diagnostic criteria for acute appendicitis was as follows: pain in the right lower quadrant; a physical examination showing tenderness in the right lower quadrant with maximum severity at the McBurney point; a blood test showing increased white blood cell (WBC) count or C-reactive protein (CRP) level or percentage of neutrophils; and ultrasound or abdominal computed tomography (CT) suggesting swelling in the appendix.

### Patient grouping

The patients were divided into 2 groups according to the hospital stay: 348 with a hospital stay ≤3 days in group A and 288 with a hospital stay >3 days in group B.

### Surgical methods

All surgeries were performed by the same group of doctors, who had 3–5 years of experience in laparoscopic appendectomy and performed more than 50 laparoscopic appendectomies every year. The patients were placed in the supine position and received general anesthesia by tracheal intubation. A 1-cm arc incision was made on the umbilicus, and a pneumoperitoneum needle was used to puncture into the abdominal cavity. A 12–14 mm Hg pneumoperitoneum was formed by inflation with carbon dioxide. The pneumoperitoneum needle was pulled out. A 10-mm trocar was used to puncture the abdominal cavity, and the inner core was pulled out to allow the entry of a laparoscope. Under laparoscopy, 1-cm and 0.5-cm small incisions were made at the anti-McBurney point and 3 cm on the pubic symphysis, and 10-mm and 5-mm trocars were placed, respectively. After changing to a left lateral position with head down and legs up, laparoscopic instruments were placed in to find and resect the appendix. A nipper was used to lift the mesenterium near the root of the appendix, and a separating plier was used to bluntly separate and penetrate the root of the appendix mesentery connected to the appendix. Either suture ligation or Hem-o-lok occlusion was performed to close the root mesentery of the appendix. The appendix root was 1igated with a 7# silk thread (Mersilk, Ethicon) 0.5 cm from the root of the appendix if the root of appendix was not edema and the root diameter with in 5mm. Otherwise it was occluded with a Hem-o-lok (Teleflex Medical, USA) 0.5 cm from the root of the appendix. The appendix was cut at a distance of 0.5 cm from the ligation position or the distal end of the Hem-o-lok. The appendix was removed by placing it in a fetching bag from the trocar in the left lower abdomen. The abdominal pelvic fluid was suctioned, and the incision was sutured.

### Postoperative care

The postoperative diet was gradually restored according to the conditions during the surgery. Ceftriaxone 2g qd + metronidazole 0.5g tid or ertapenem 1g qd were used for intravenous antibiotic treatment according to the severity of infection. The wound dressing was changed 2–3 days after the surgery. The patients were discharged if the blood test results were basically normal (WBC<10x109/L, N%<85% and CRP<50mg/L), the patients tolerated semi-liquid food, they had no fever or wound infection, and they had controlled pain.

### Data collection

The following indicators were monitored: sex, age, disease onset time (time from onset to admission), nausea, vomiting, diarrhea, peritonitis, comorbidities, and history of appendicitis; preoperative T, WBC count, percentage of neutrophilic granulocytes, and CRP level; time from diagnosis to surgery. appendix diameter, appendicolith, and ascites in ultrasound or CT; surgical time (the surgery start time was the time of skin incision, and the end time was the time the anesthesia intubation was removed), intraoperative blood loss (the volume of blood infiltrating into a gauze was calculated by weighing the gauze infiltrated with water and calculating the volume of water), intraoperative adhesions or effusions, and stump closure methods; convert to open appendectomy, appendix pathology and antibiotic treatment schemes and hospital stay.

### Statistical analysis

All the results were analyzed using SPSS 16.0 software (IBM, USA). Continuous variables were compared using the Mann–Whitney test, while the chi-square test was used for comparing the frequency data. Binary logistic regressions were performed using the hospital stay (cutoff = 3 days) as the outcome, and related factors were investigated. A P <0.05 indicated a statistically significant difference.

## Results

The surgeries were successfully completed, with 37 cases (15 cases in group A and 22 cases in group B) converted into open surgeries. Group A comprised 169 male and 179 female patients aged 37.10 ± 13.52 years, while group B comprised 142 male and 146 female patients aged 42.94 ± 15.57 years. Compared with patients in group A, patients in group B had longer disease onset time (21.36 ± 16.56 h vs 32.52 ± 27.99 h, P < 0.01), longer time from diagnosis to surgery (8.63 ± 7.29 h vs 10.70 ± 8.47 h, P < 0.01), longer surgical time (64.09 ± 17.24 min vs 86.19 ± 39.96 min, P < 0.01), higher incidence of peritonitis (52.9% vs 74%, P < 0.01), comorbidities (12.4% vs 20.5%, P < 0.01), appendicolith (27.6% vs 41.7%, P < 0.01), ascites before the surgery (13.8% vs 22.9%, P < 0.01), intraoperative adhesions or effusions (56% vs 80.2%, P < 0.01), higher temperature (37.11 ± 0.64°C vs 37.54 ± 0.90°C, P < 0.01), higher preoperative WBC count (13.06 ± 3.39 × 109/L vs 14.21 ± 4.54 × 109/L, P = 0.04), higher preoperative CRP level (18.99 ± 31.72 mg/L vs 32.46 ± 46.68 mg/L, P < 0.01), higher appendix diameter (10.22 ± 2.59 mm vs 11.26 ± 3.23 mm, P < 0.01), more intraoperative blood loss (9.36 ± 7.29 mL vs 13.74 ± 13.49 mL, P < 0.01), higher rate of using Hem-o-lok for stump closure (30.7% vs 38.5%, P = 0.04), higher ratio of complicated appendicitis (9.5% vs 45.8%, P < 0.01), and higher rate of using ertapenem for antibiotic treatment after the surgery (4.3% vs 21.5%, P < 0.01). No significant differences were found between the two groups in sex, presence or absence of symptoms, presence or absence of history of appendicitis, temperature, preoperative neutrophil percentage, neutrophil percentage on the second postoperative day, CRP level on the second postoperative day, or postoperative wound infection rate. None of the patients in the two groups had stump fistula within 3 months after the surgery, as shown in Table 1. Neither mortality nor postoperative abdominal abscess was detected in the two groups.

**Table 1:** Comparison of characteristics and outcomes between the two groups.

	Group A (hospital stay≤3 days)	Group B (hospital stay>3d)	*p*
	*n* = 348	*n* = 288	
**Age (year, mean ± SD)**	37.10 ± 13.52	42.94 ± 15.57	<0.01
**Sex, male (*n*, %)**	169, 48.6%	142, 49.3%	0.85
**Disease onset time (hours, mean ± SD)**	21.36±16.56	32.52±27.99	<0.01
**Nausea, vomiting or diarrhea (*n*, %)**	264, 75.9%	203, 70.5%	0.13
**Peritonitis (*n*, %)**	184, 52.9%	213, 74%	<0.01
**Comorbidities (*n*, %)**	43, 12.4%	59, 20.5%	<0.01
**History of appendicitis (*n*, %)**	50, 14.4%	39, 13.5%	0.77
**Temperature (°C, mean ± SD)**	37.11±0.64	37.54±0.90	<0.01
**Preoperative white blood cell count (10**^**9**^**/L, mean ± SD)**	13.06±3.39	14.21±4.54	0.04
**Preoperative CRP level (mg/L, mean ± SD)**	18.99±31.72	54.14±66.13	<0.01
**Preoperative neutrophil percentage (%, mean ± SD)**	81.24±9.23	82.96±8.48	0.11
**Time from diagnosis to surgery (h, mean ± SD)**	8.63±7.29	10.70±8.47	<0.01
**Appendix diameter (mm, mean ± SD)**	10.22±2.59	11.26±3.23	<0.01
**Appendicolith (*n*, %)**	96, 27.6%	120, 41.7%	<0.01
**Ascites before surgery (*n*, %)**	48, 13.8%	66, 22.9%	<0.01
**Intraoperative adhesions or effusions (*n*, %)**	195, 56%	231, 80.2%	<0.01
**Surgical time (min, mean ± SD)**	64.09±17.24	86.19±39.96	<0.01
**Blood loss (ml, mean ± SD)**	9.36±7.29	13.74±13.49	<0.01
**Stump closure**			0.04
**Silk ligation (*n*, %)**	241, 69.3%	177, 61.5%	
**Hem-o-lok (*n*, %)**	107, 30.7%	111, 38.5%	
**Convert into open appendectomy (*n*, %)**	15, 4.3%	22, 7.6%	0.07
**Appendix pathology (*n*, %)**			<0.01
**Uncomplicated (*n*, %)**	315, 90.5%	156, 54.2%	
**Complicated (*n*, %)**	33, 9.5%	132, 45.8%	
**Antibiotics (*n*, %)**			<0. 01
**Cephalosporins + metronidazole (*n*, %)**	333, 95.7%	226, 78.5%	
**Ertapenem (*n*, %)**	15, 4.3%	62, 21.5%	
**White blood cell count on the second postoperative day (10**^**9**^**/L, mean ± SD)**	8.46±3.11	8.08±3.13	0.04
**Neutrophil percentage on the second postoperative day (%, mean ± SD)**	69.73±12.42	82.96±8.48	0.56
**CRP level on the second postoperative day (mg/L, mean ± SD)**	68.18±62.32	75.55±62.37	0.07
**Postoperative wound infection (*n*, %)**	13, 3.7%	11, 3.8%	0.96
**Stump fistula 3 months after surgery (*n*, %)**	0, 0.0%	0, 0.0%	NA

Logistic regression was performed and demonstrated that age [odds ratio (OR) = 1.021; 95% confidence interval (CI) = 1.007–1.036], peritonitis (OR = 1.603; 95% CI = 1.062–2.419), preoperative WBC count (OR = 1.084; 95% CI = 1.025–1.046), preoperative CRP level (OR = 1.010; 95% CI = 1.005–1.015), time from diagnosis to surgery (OR = 1.043; 95% CI = 1.015–1.072), appendicolith (OR = 1.852; 95% CI = 1.222–2.807), complicated appendicitis (OR = 3.536; 95% CI = 2.132–5.863), surgical time (OR = 1.025; 95% CI = 1.016–1.034), use of Hem-o-lok for stump closure (OR = 1.894; 95% CI = 1.257–2.852), and use of ertapenem for antibiotic treatment (OR = 3.076; 95% CI = 1.483–6.378) were the risk factors for a prolonged hospital stay, as shown in Table 2.

**Table 2:** Factors associated with hospital stay after logistic regression.

Factor	Wald	*p*	OR	95%CI
**Age**	8. 567	<0. 01	1. 021	1. 007–1. 036
**Peritonitis**	5. 045	0. 03	1. 603	1. 062–2. 419
**Preoperative white blood cell count**	8. 095	<0. 01	1. 084	1. 025–1. 046
**Preoperative CRP level**	15. 401	<0. 01	1. 010	1. 005–1. 015
**Time from diagnosis to surgery**	9. 401	<0. 01	1. 043	1. 015–1. 072
**Appendicolith**	8. 428	<0. 01	1. 852	1. 222–2. 807
**Appendix pathology (Complicated appendicitis)**	23. 948	<0. 01	3. 536	2. 132–5. 863
**Surgical time**	27. 704	<0. 01	1. 025	1. 016–1. 034
**Stump closure (Hem-o-lok)**	9. 344	<0. 01	1. 894	1. 257–2. 852
**Antibiotics (Ertapenem)**	9. 120	<0. 01	3. 076	1. 483–6. 378

## Discussion

The shorter hospital stay can lead to less cost and a higher percentage of bed utilization. Therefore, it is important to investigate its influencing factors.

This study demonstrated that the length of hospital stay increased with the age of patients. The patients in the group with a prolonged hospital stay were older than the patients in other groups. Elder people are at higher risk of malnutrition which will lead to a higher risk of post operation infection. Therefore, the elderly patients took more time to return to normal after the surgery.

Preoperative high WBC count and CRP level and peritonitis indicated severe appendicitis. Regarding the AIR scores, the WBC count >15 × 109/L and the CRP level>50 mg/L were used as the criteria for the diagnosis of severe abdominal infection. The accuracy rate of these criteria in distinguishing severe acute abdominal infection was as high as 85% [17]. The postoperative recovery time will be prolonged due to more inflammatory exudation and longer absorption time with severe infection. The CRP level of acute perforated appendicitis is higher [18]. Hence, the preoperative CRP level can better reflect the high possibility of complicated appendicitis compared with the preoperative WBC count. Complicated appendicitis always leads to severe infection. In addition, the massive separation and the residual purulent exudation in the abdominal cavity during the surgery can also lead to prolonged postoperative recovery time, as shown in the present study.

The key to the successful treatment of acute appendicitis is timely surgery after diagnosis. How long it will be safe from diagnosis to surgery is still controversial. The hospital stays of patients with the diagnosis to surgical time more than 24 h was significantly longer [18]. Moreover, a prolonged hospital stay was attributable to the time from diagnosis to the surgery of 12–24 h [19]. However, a meta-analysis based on 152,314 cases in 45 published studies showed that the time from diagnosis to surgery within 24 h would not prolong the hospital stay [20]. The present study showed that the time between diagnosis and surgery was the risk factor for the prolongation of the hospital stay. However, the cut-off point requires further analysis.

The present study demonstrated that patients with appendicolith had a significantly longer hospital stay. Appendicolith can cause the obstruction of the appendiceal lumen, contributes to bacterial overgrowth and continued secretion of mucus, leading to intraluminal distention and increased wall pressure. Subsequent impairment of lymphatic and venous drainage leads to mucosal ischemia. These processes in combination promote a localized inflammatory process that may progress to gangrene and perforation. Appendicolith was found to be an independent risk factor for appendiceal perforation [21, 22]. Perforated appendicitis leads to severe infection, and severe infection leads to prolonged recovery time as discussed earlier.

The surgeons involved in the study were all experienced doctors. Therefore, the extension of the surgical time was attributable to the severity of infection and abdominal adhesion. Severe adhesion leads to more severe trauma after extended dissection and more local exudation, eventually leading to prolonged postoperative recovery time. Therefore, the present study demonstrated that prolonged surgical time was a risk factor for the prolongation of hospital stay.

The treatment of the appendix stump is the key to a successful surgery. The commonly used methods are silk ligation, Endoloop ligation, Hem-o-lok vascular clamp occlusion, and endo-gastrointestinal anastomosis (Endo-GIA) stapler cutting and closure [7–11, 14–16, 23–28]. Vascular clamps were associated with shorter surgical time and were cheaper [12, 13, 15, 16]. However, they had increased costs compared with simple ligation. The multiple logistic regression analysis suggested that securing the stump with Hem-o-lok was an independent factor related to a prolonged hospital stay. The discharge criteria included controlled pain, and hence were related to the difference in hospital stay. The Hem-o-lok vascular clamp was stiff, and therefore it might stimulate the surface of the bowel during peristalsis of the intestine, causing abdominal pain in the short term after surgery. Sadat-Safvi et al. [15] found that the incidence of postoperative abdominal pain after vascular clamp treatment of appendix stump was as high as 76%. However, a silk thread is soft and does not cause similar irritation to the intestinal tract. Therefore, a patient is likely to have less postoperative pain.

The main pathogens causing appendicitis are *Escherichia coli* and anaerobes. Ceftriaxone, metronidazole, and ertapenem are commonly used antibiotics for appendicitis [29]. Ertapenem and ceftriaxone plus metronidazole are not different in treating acute appendicitis [30, 31]. The present study demonstrated that the use of ertapenem was a risk factor for a prolonged hospital stay. Most of the patients treated with ertapenem had a severe infection or complicated appendicitis. Selection bias might influence the results, which requires further exploration.

## Limitations

This study had some limitations. It was a single-center study with a small sample size. Therefore, the results need to be confirmed in larger groups of patients. Further, it was a retrospective study, and the patients were not randomly assigned to their treatment groups, leading to some bias. The findings of the multivariate analysis should be confirmed with a randomized controlled trial.

In summary, patients with appendicitis were older and had peritonitis, higher preoperative WBC count or CRP level, longer time from diagnosis to surgery, appendicolith, and complicated appendicitis, which might predict a prolonged hospital stay. Shorter surgical time and the use of silk ligation for stump closure and cephalosporins + metronidazole for antibiotic treatment might be better choices to obtain shorter hospital stay.

## Supporting information

S1 File (XLS)
